# Long non-coding RNA ATB promotes human non-small cell lung cancer proliferation and metastasis by suppressing miR-141-3p

**DOI:** 10.1371/journal.pone.0229118

**Published:** 2020-02-24

**Authors:** Guojie Lu, Yaosen Zhang

**Affiliations:** Department of thoracic surgery, Guangzhou Panyu Central Hospital, Guangzhou, PR China; Universitat des Saarlandes, GERMANY

## Abstract

Long noncoding RNA activated by transforming growth factor-β (lncRNA-ATB) plays a critical role in progression of several cancers. In this study, lncRNA-ATB was significantly up-regulated in NSCLC tissues and cell lines, and high lncRNA-ATB expression indicated poor prognosis. Knockdown of lncRNA-ATB suppressed NSCLC cell growth, colony formation, migration, invasion and reversed epithelial-mesenchymal transition. *In vivo* study showed that silencing lncRNA-ATB inhibited tumor growth. Further mechanism studies demonstrated that lncRNA-ATB was a target of miR-141-3p. MiR-141-3p expression was negatively related to lncRNA-ATB expression in NSCLC tissues. These results suggested that inhibiting lncRNA-ATB might be an approach for NSCLC treatment.

## Introduction

Lung cancer is one of the most frequently diagnosed tumors, which ranks the third most common in the world. Non-small cell lung cancer (NSCLC) is the most common type of lung cancer, accounting for about 80% of all cases [1]. The overall 5-year survival rate of NSCLC patients still remains poor, despite substantial advances achieved in the treatment [2–4]. Although mounting evidence has documented that alterations in many tumor-suppressor genes and oncogenes are associated with NSCLC, the molecular and genetic bases of NSCLC remain largely unknown [5–7]. Undoubtedly, better understanding of the underlying pathological mechanisms will contribute to develop more effective therapeutic strategies, thereby improving the clinical outcome of NSCLC patients. Recently, the regulation of the non-protein-coding genome in normal physiology and the pathogenesis of diseases including NSCLC have attracting growing intention [8, 9].

Long non-coding RNA (lncRNA) comprises a class of transcripts with more than 200 nucleotides without protein-coding ability [10]. Accumulating evidences revealed that many lncRNAs can regulate tumor cells proliferation, cell cycle, apoptosis, drug resistance, migration, invasion, and histone modification. For example, Long noncoding RNA SNHG1 promotes NSCLC viability, proliferation, migration, and invasion by up-regulating MTDH via sponging miR-145-5p [11]. LncRNA-LET expression was inversely associated with advanced tumor stages and poorer overall survival of NSCLC patients. Overexpression of lncRNA-LET in NSCLC H292 cells significantly suppressed cell proliferation, migration and invasion, and promoted cell cycle arrest and apoptosis, pointing to a tumor-suppressive role for lncRNA-LET in NSCLC [12]. Besides these examples, a variety of novel lncRNAs remain to be elucidated and characterized.

In the current study, we investigated how Long noncoding RNA activated by transforming growth factor-β (lncRNA-ATB) contributes to the progression of NSCLC and explored the underlying mechanisms.

## Materials and methods

### Tissue samples

A total of 68 paired lung adenocarcinoma tissues confirmed by histopathology and adjacent normal tissues (>3 cm away from tumor) were obtained from patients who received surgical resection of lung adenocarcinoma between 2011 and 2013 in our hospital. None of the patients received radiotherapy or chemotherapy before surgery. The samples were immediately frozen in liquid nitrogen and further stored at -80°C until total RNA and protein extraction. The study was approved by the Institutional Review Boards of Guangzhou Panyu District Central Hospital (license number of medical ethics committee: ZZ-BX-2011213, Date: 2011.2.15). Assigning written informed consent was obtained from all subjects.

### Cell culture

Five NSCLC cell lines (A549, NCI-H292, SPC-A-1, NCI-H460, and NCI-H1703) and human bronchial epithelial (16HBE) cell were obtained from the Cell Bank of Type Culture Collection (Chinese Academy of Sciences, Shanghai, China). All cells were cultured in Dulbecco’s modified Eagle's medium (DMEM; Hyclone, Logan, UT, USA), along with 10% fetal bovine serum (Invitrogen, Grand Island, NY, USA), 100 μg/mL streptomycin and 100 U/mL penicillin. Cells were kept at 37°C, 5% CO_2_ in an incubator.

### RNA isolation and qRT-PCR

Total RNA was extracted using Trizol reagent (Invitrogen), and cDNA was synthesized by using a Reverse Transcription Kit (Takara, Dalian, China). The expression of lncRNA-ATB was quantified on ABI 7500 real-time PCR system, and GAPDH was used as an internal control. The expression of miR-141-3p was quantified by using TaqMan miRNA assays in accordance with the manufacturer’s instructions (Applied Biosystems, Foster City, CA, USA), and U6 was used as an internal control. Fold change in gene expression was calculated by using the 2^-ΔΔCt^ method. The sequence of primers applied in qRT-PCR were shown as follows: lncRNA-ATB sense: 5'-TCTGGCTGAGGCTGGTTGAC-3',

lncRNA-ATB anti-sense: 5'-ATCTCTGGGTGCTGGTGAAGG-3',

GADPH sense: 5'-GGTCGGAGTCAACGGATTTG-3',

GADPH anti-sense: 5'-ATGAGCCCCAGCCTTCTCCAT-3',

miR-141-3p sense: 5'-CGTCGCTAACACTGTCTGGTAA-3',

miR-141-3p anti-sense: 5'-GTGCAGGGTCCGAGGTATTC-3',

U6 sense: 5'-GCTTCGGCAGCACATATACT-3',

U6 anti-sense: 5'-GGTGCAGGGTCCGAGGTATT-3'.

### Transfection

Three individual lncRNA-ATB siRNAs (si-lncRNA-ATB) and scrambled negative control siRNA (si-NC) were purchased from Ribo BioCoLTD (Guangzhou, China). All transfection reactions were performed by using Lipofectamine 2000 (Invitrogen), according to the manufacturer’s protocol. After transfection for 48 h, cells were further used in the relevant experiments.

### Cell counting kit-8 assay

The cell counting kit-8 (CCK-8) assay was conducted according to the manufacturer’s protocols (CCK-8; Dojindo, Japan). The two experimental groups (si-NC and si-lncRNA-ATB) of A549 and SPC-A-1 cells were seeded into 96-well plates at a density of 5000 cells/well and then cultured for 72 h in succession. 10 μl of CCK- 8 solutions was added to each well, and incubated for 3 h at 37°C. The optical density (OD) was measured at 450 nm (OD450).

### Colony formation assay

Colony formation assay was performed to detect the clone formation ability and compare the difference among the two experimental groups (si-NC and si-lncRNA-ATB) of A549 and SPC-A-1 cells respectively. Cells were seeded into 6-well plates at a density of 500 cells per well, after consecutively cultured for 14 days at 37°C, the cells were stained with crystal violet and then the colonies were counted in each dish to estimate the plating efficiency among different groups. Visible formed colonies with >50 cells were counted by light microscopy.

### Scratch assay

Cells were seeded in 6-well plates and were transfected with si-NC or si-lncRNA-ATB. After cultured overnight, scratches were made to the cell monolayers with a 200 μl pipette tip. Then the cells were washed twice with PBS to remove the floating cells. Thereafter, total medium supplemented with 1 μg/ml Mitomycin C was added to the wells. Photographs were taken after 0 and 48 h after the scratch.

### Invasion assays

200 μL of 48 h post-transfected cells suspension with the concentration as 2×10^5^ cells/mL were seeded into the upper chamber (Costar, USA) with 8 μm pores. Meanwhile, 500 μL of DMEM containing 10% FBS was put into the lower chamber. And then the plates were incubated in the incubator for 48 h. After incubation, the medium was removed from the upper chamber and each group cells in the upper chamber were scraped off with a cotton swab. The number of cells invading the matrigel in each group was fixed with 4% formaldehyde for staining with 0.1% crystal violet. Then we counted cells in three randomly selected fields under an inverted microscope (200× magnification).

### Western blot analysis

Total protein was isolated using RIPA lysis buffer containing protease inhibitors. Protein concentrations were measured by Bradford dye (Bio-Rad). Protein (50μg of each sample) were separated by 10% SDS-PAGE and transferred electrophoretically onto PVDF membranes (Millipore, Billerica, MA, USA). Then, the membranes were blocked in 5% skim milk for 1 h, washed three or four times with Tris-buffered saline containing 20% Tween 20 (TBST) at room temperature, and incubated with the primary antibodies at 4°C overnight. Following washing with TBST, the membranes were then immersed in secondary antibody (goat anti-rabbit IgG, 1:1000, Santa Cruz Biotechnology) at room temperature for 1 h. After TBST wash, the immunoreactivity was visualized by ECL solutions (Thermo Pierce). β-actin (Santa Cruz Biotechnology) served as an endogenous control.

### Dual luciferase reporter assay

The lncRNA-ATB sequence containing wild-type (WT) or mutant (MUT) binding sites of miR-141-3p was inserted into the pmiR-RB-REPORT^™^ (RiboBio, Guangzhou, China). A549 cells were co-transfected with ATB-WT or ATB-Mut reporter plasmids and miR-141-3p mimics or negative control (miR-NC) for 48 h by using Lipofectamine 3000. Relative luciferase activity was detected by dual-glo luciferase reporter system (Promega, Madison, WI, USA).

### RNA-binding protein immunoprecipitation assay (RIP)

Imprint RNA Immunoprecipitation kit was purchased from Sigma-Aldrich (St. Louis, MO, USA). Ago2 and IgG antibodies were obtained from Abcam (Cambridge, MA, USA). RNA-binding protein immunoprecipitation assay was conducted by using Imprint RNA Immunoprecipitation kit and the Ago2 antibody according to the manufacturer’ protocol. The IgG antibody was used as negative control. qRT-PCR was applied to determine the enrichment of ATB and miR-141-3p in immunoprecipitated RNA.

### RNA pulldown assay

Biotin-labeled miR-141-3p (miR-141-3p-Bio) and Biotin-labeled miR-NC (miR-NC-Bio) were commercially synthesized by Guangzhou RiboBio Co.,LTD (Guangzhou, China). A549 cells were transfected with miR-NC-Bio or miR-141-3p-Bio. At 48 h post-transfection, A549 cells were subjected to RNA pulldown assay by using Pierce^TM^ Magnetic RNA Protein Pull-down Kit (Thermo Fisher Scientific) in accordance with the manufacturer’s specification. The enrichment of lncRNA-ATB was assessed with qRT-PCR assay.

### Animal work

Male BALB/c nude mice aged 5 weeks were purchased from Slac Laboratory Animal Center (Shanghai, China) and maintained under specific pathogen free (SPF) condition in the animal care facility. A549 cells transfected with si-lncRNA-ATB or si-NC were harvested and resuspended in phosphate-buffered saline (PBS), and the cell concentration was adjusted to 1×10^8^ cells/ml. Then, 100 μl of suspending cells transfected with si-lncRNA-ATB was subcutaneously injected into right side of the posterior flank of the nude mouse. Tumor diameter was recorded using a vernier caliper every three days. Tumor volume was calculated follows formula: tumor volume (mm^3^) = (length×width^2^)/2. Mice were euthanatized on day 21, and the tumors were excised and weighed. All animal experiments were authorized by the Experimental Animal Ethics Committee of Guangzhou Panyu Central Hospital (Guangzhou, China).

### Methods of sacrifice

Carbon dioxide euthanasia is used for all animals utilized in this research.

### Steps to minimize pain and distress

In this study, we haven’t found the mice has showed any obvious abnormalities, such as bodyweight loss, appetite loss, or infection of organs. All of the mice used in this study were euthanatized at the experiment endpoint.

If animals became ill, different steps will be taken to minimize pain and distress. For example, the claws of mice were hurt because of struggling hardly when mice are taken from the cage for drug administration. Firstly, the wound must be disinfected. Then the claws were bound up by gauze. Last but not least, mice were taken minor anesthesia to relieve the pain if applicable.

### Statistical analysis

Data were expressed as the mean ± standard deviation (SD). One-way analysis of variance (ANOVA) was used to analyze the significance among more than three groups. And Student t test was applied to evaluate the significance between two groups. Survival analysis was assessed using Kaplan-Meier method. The correlations between lncRNA-ATB expression and clinicopathologic factors were evaluated using Chi-Square test. Statistical significance was set at *P* < 0.05.

## Results

### LncRNA-ATB is up-regulated in human NSCLC tissues and cell lines

**Fig 1:**
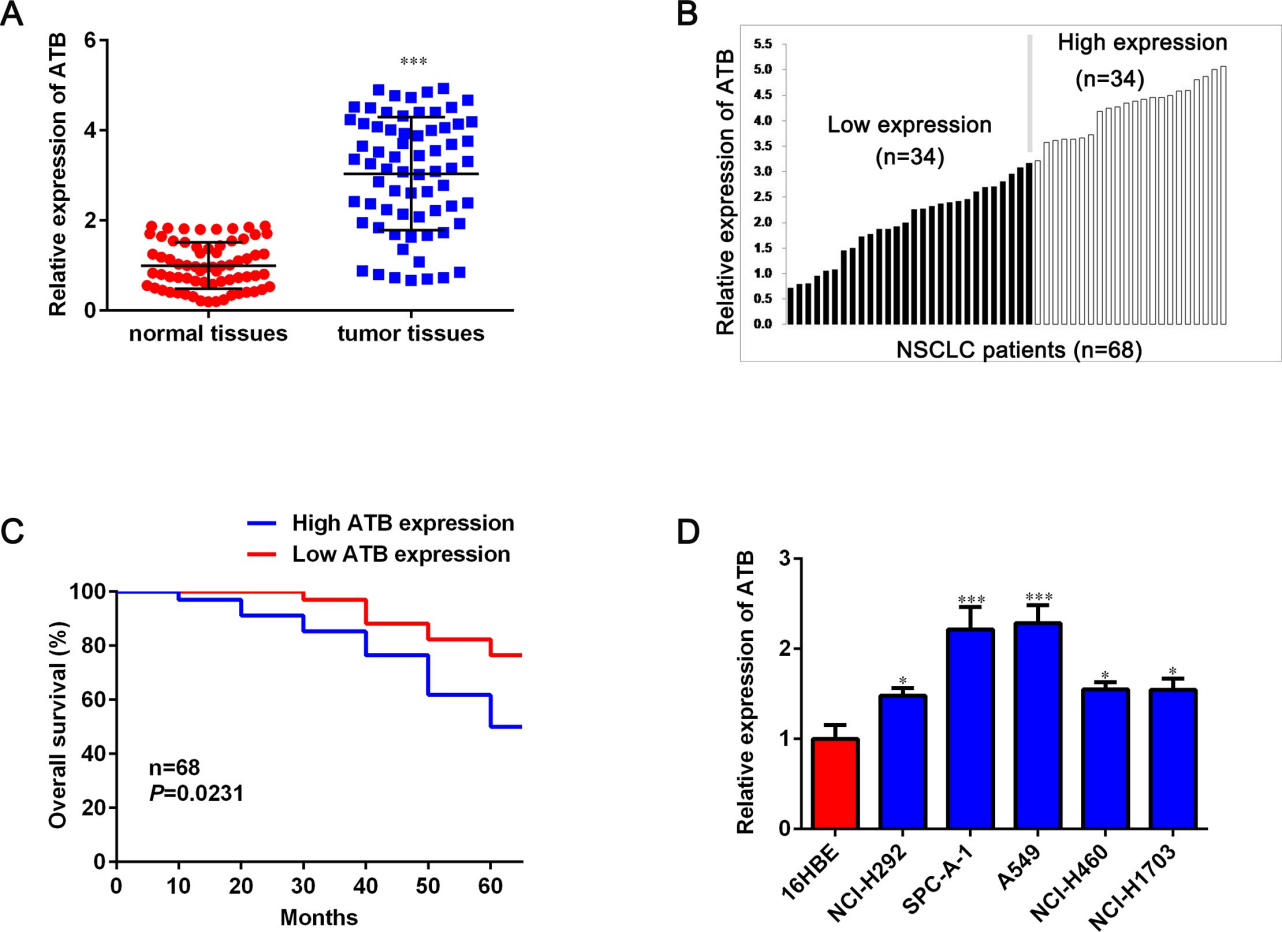
Expression of lncRNA-ATB in NSCLC tissues and its association with clinical factors. A. The lncRNA-ATB expression level in 68 NSCLC tissues and paired adjacent normal tissues was detected by qRT-PCR and normalized to GAPDH expression. B. Samples were divided into a high lncRNA-ATB expression group (34 pairs) and a low lncRNA-ATB expression group (34 pairs) based on the median level. C. The relationship between levels of lncRNA-ATB and overall survival was analyzed by Kaplan-Meier survival analysis. D. RNA levels of lncRNA-ATB in NSCLC cell lines and normal cell line. Data are shown as mean ± SD. *P < 0.05, **P < 0.01 and ***P < 0.001.

LncRNA-ATB expression in 68 pairs of NSCLC lung tissues and paired adjacent normal lung tissues was determined by using qRT-PCR. As shown in Fig 1A, lncRNA-ATB expression was significantly increased in the NSCLC tissues when compared with that in the adjacent normal tissues. Furthermore, we divided the 68 patients into high and low expression groups based on the median lncRNA-ATB level to explore correlations between lncRNA-ATB expression and clinicopathological features (Fig 1B). The relationship of lncRNA-ATB with various clinical features of NSCLC was analyzed and is summarized in Table 1. It could be informed that high expression of lncRNA-ATB was related to large tumor size (*P* < 0.05), poorly differentiated (*P* < 0.05) and tumor metastasis (*P* < 0.05). However, we failed to observe any relationship between lncRNA-ATB and other clinicopathological characteristics (gender, age and smoking). The Kaplan-Meier analyses were further conducted to explore the correlation of lncRNA-ATB expression with overall survival of NSCLC patients. The Kaplan-Meier survival analysis showed that the high lncRNA-ATB was associated with poor prognosis in patients with NSCLC (*P* < 0.05, Fig 1C). We then determined lncRNA-ATB expression in NSCLC cell lines NCI-H292, A549, SPC-A-1, NCI-H460 and NCI-H1703, and human bronchial epithelial (16HBE) cells. All five NSCLC cell lines had higher lncRNA-ATB expression than the normal 16HBE cell line (Fig 1D).

**Table 1:** Correlation between ATB expression and clinical.

Parameters	Relative ATB expression level		*P* value
	High (n = 34)	Low (n = 34)	
Sex			0.7930
Men	24	23	
Women	10	11	
Age			0.6270
≤ 60	19	17	
>60	15	17	
Smoking			0.1428
Non-smoking	12	18	
Smoking	22	16	
Tumor size			0.0290*
≤ 3 cm	13	22	
> 3 cm	21	12	
Histological grade			0.0034**
Well	9	21	
Poor	25	13	
Metastasis			0.0001***
Negative	8	24	
Positive	26	10	

### Knockdown of lncRNA-ATB inhibited cellular proliferation

**Fig 2:**
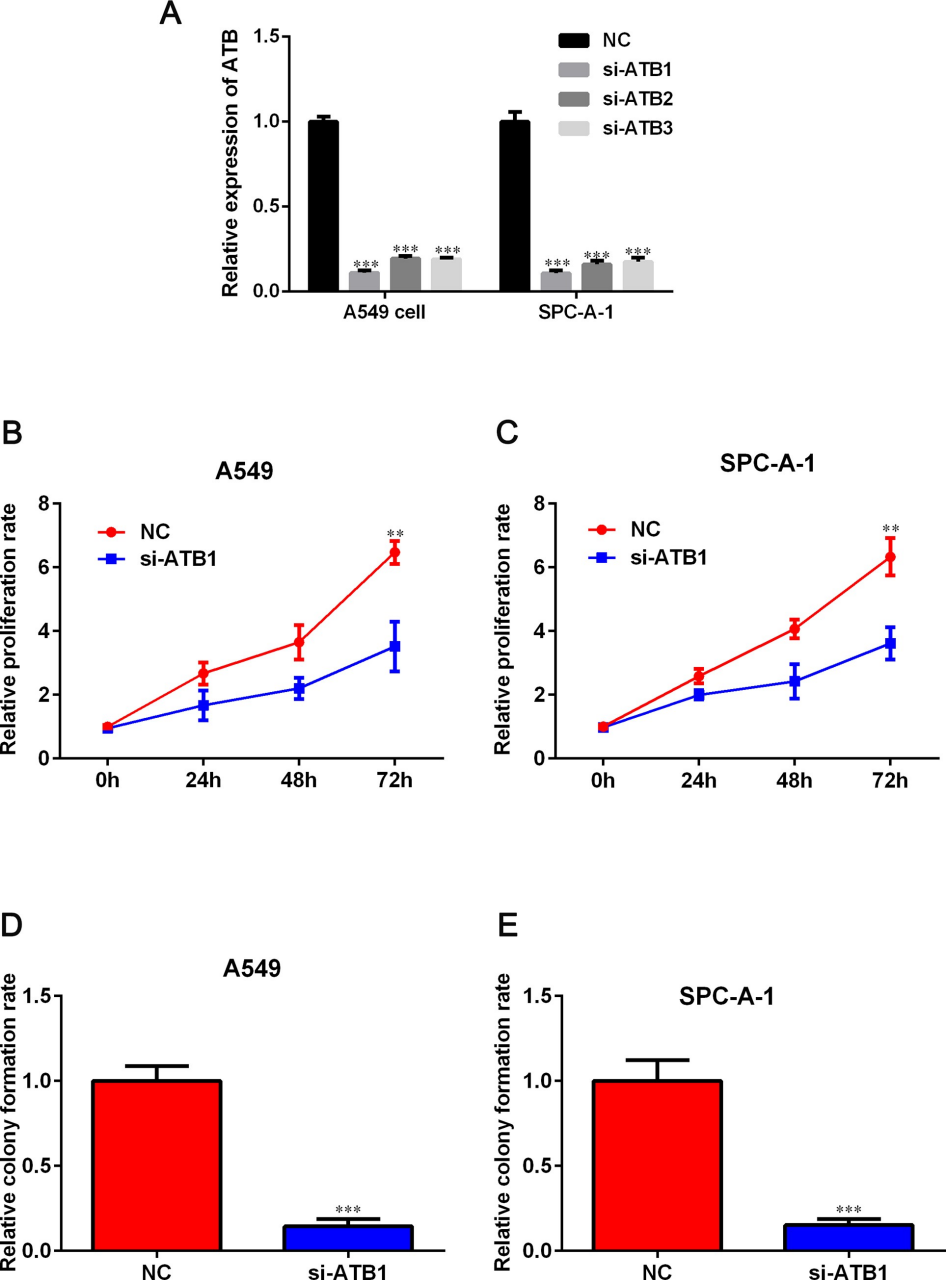
Silencing of lncRNA-ATB expression inhibited growth in NSCLC cells. A. Quantification of lncRNA-ATB expression level after si-lncRNA-ATB transfection into the A549 and SPC-A-1 cell lines. B, C. Cell growth was measured by the CCK-8 assay after A549 and SPC-A-1 cells after transfected with si-lncRNA-ATB. D, E. Colony formation assay was performed to detect the proliferation ability of A549 and SPC-A-1 cells after transfected with si-lncRNA-ATB. Data are expressed as mean ± SD. *P < 0.05, **P < 0.01 and ***P < 0.001 versus NC group.

3 siRNAs were synthesized and transfected into A549 and SPC-A-1 cells to silence lncRNA-ATB expression. qRT-PCR results showed that lncRNA-ATB expression was significantly decreased by siRNA 1. Therefore, siRNA 1 was selected in the further experiments (Fig 2A). CCK-8 assay analysis revealed that downregulation of lncRNA-ATB significantly repressed the proliferation of both A549 and SPC-A-1 cells (*P* < 0.05, Fig 2B and 2C). Additionally, colony formation assays showed that the clonogenic ability of A549 and SPC-A-1 cells was decreased after si-lncRNA-ATB interference (Fig 2D and 2E).

### Silencing of lncRNA-ATB inhibits metastases and EMT of NSCLC cells

**Fig 3:**
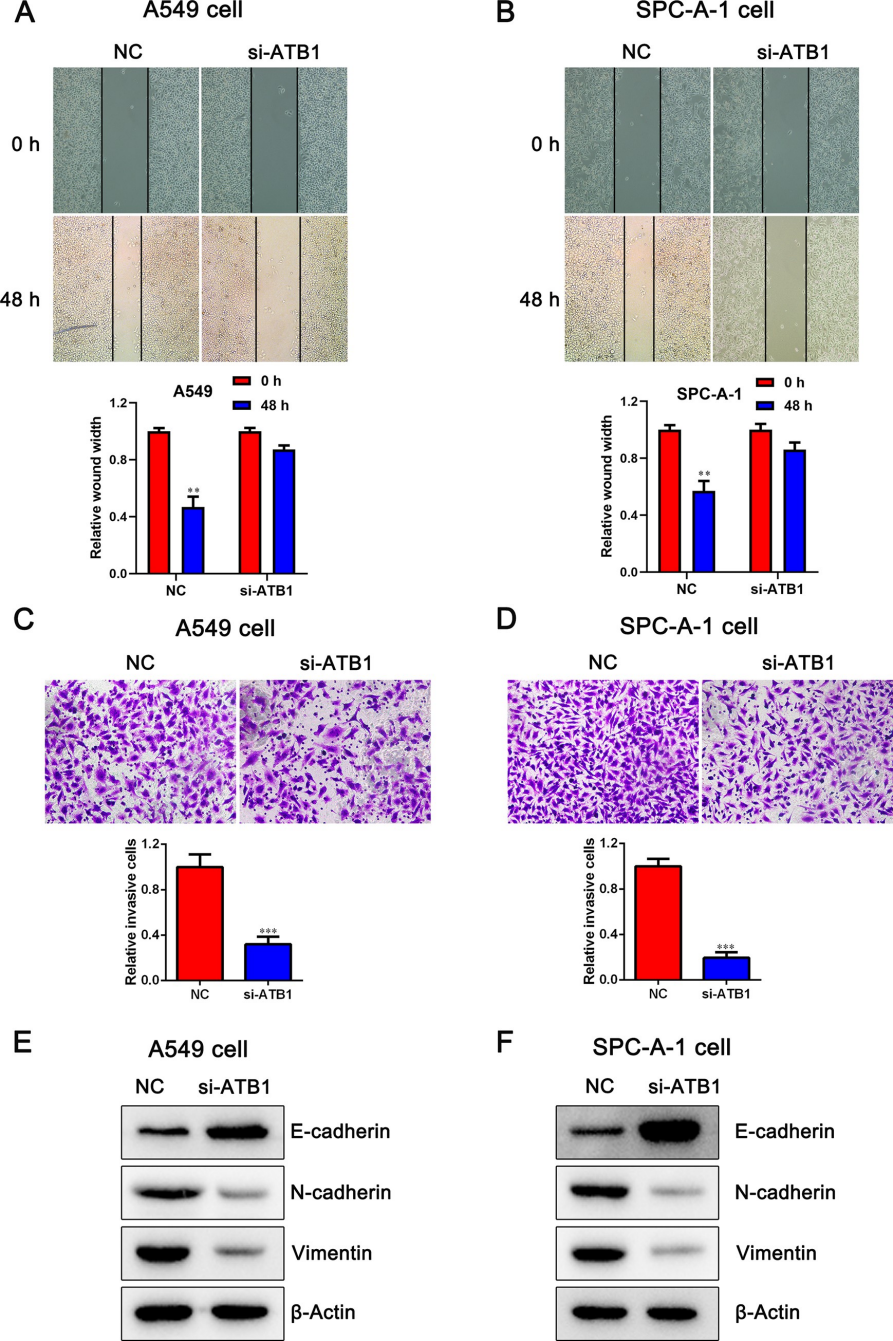
Effects of lncRNA-ATB on migration and invasion in NSCLC cells. A, B. Detection for cell migration ability of A549 and SPC-A-1 cells after transfected with si-lncRNA-ATB. C, D. Transwell chamber assay was employed to examine the invasion ability of A549 and SPC-A-1 cells after transfected with si-lncRNA-ATB. E, F. Western blot assay showed the protein level of EMT markers in A549 and SPC-A-1 cells after transfected with si-lncRNA-ATB. Data are presented as mean ± SD. ***P < 0.001 versus NC group.

We further performed the scratch and invasion assay to determine the malignant characteristics of lncRNA-ATB. The scratch assay showed that the migratory ability of A549 and SPC-A-1 cells was inhibited as indicated by the decrease in migrated cells, after transfected by si-lncRNA-ATB (*P* < 0.05, Fig 3A and 3B). Moreover, the Transwell assay produced the same results as the wound-healing assay with regard to changes in cell migration ability (*P* < 0.05, Fig 3C and 3D). We further evaluated whether lncRNA-ATB can promote EMT processes in NSCLC cancer. The data suggested that the depletion of lncRNA-ATB led to the upregulation of E-cadherin and the downregulation of N-cadherin and Vimentin, which indicates that lncRNA-ATB may induce EMT in the NSCLC cells (Fig 3E and 3F).

To further minimize confounding by off-target effects of lnc-ATB siRNA, we performed functional assays of a second siRNA. As shown in S1 Fig and S2 Fig, the second siRNA of lnc-ATB (si-ATB2) has similar effects compared with the first siRNA (si-ATB1), indicating that the silencing specificity of lnc-ATB siRNA is well.

### LncRNA-ATB is a target of miR-141-3p

**Fig 4:**
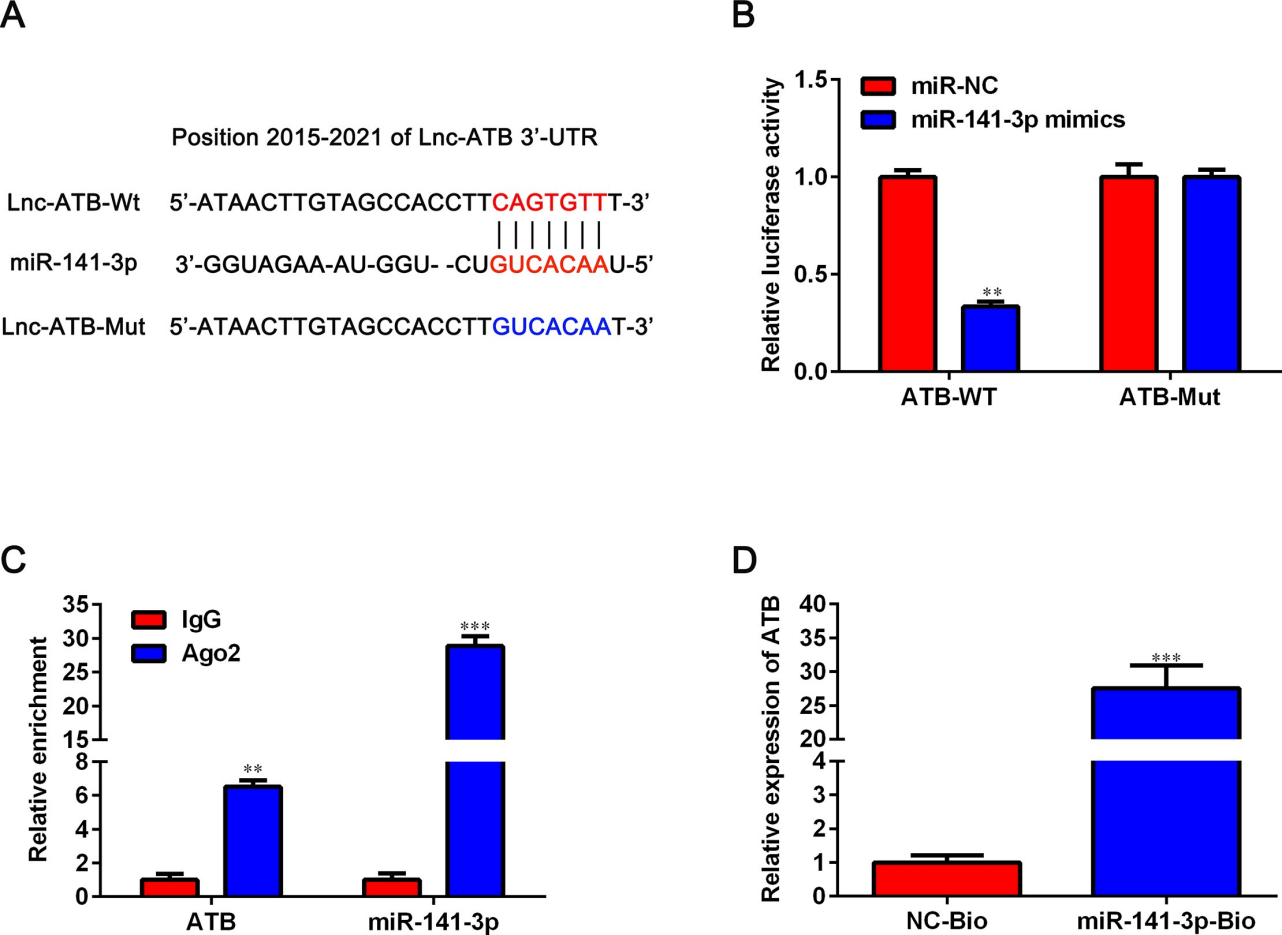
Identification of lncRNA-ATB as a target of miR-141-3p. A. The complementary sequence of miR-141-3p in lncRNA-ATB was predicted by Starbase (http://starbase.sysu.edu.cn/). B. Relative Firefly/Renilla luciferase activity mediated by ATB-WT or ATB-Mut luciferase reporter plasmids upon transfection with miR-141-3p mimics or negative control was determined by dual-luciferase assay. C. RIP experiment was conducted to assess the endogenous binding between lncRNA-ATB and miR-141-3p in A549 cells by using Ago2 antibody. D. RNA pull-down assay followed by qRT-PCR to assay lncRNA-ATB endogenously associated with miR-141-3p. Data are shown as mean ± SD. **P < 0.01 and ***P < 0.001 versus NC group or IgG group.

Bioinformatics analysis by Starbase (http://starbase.sysu.edu.cn/) revealed that there was a putative biding site between lncRNA-ATB and miR-141-3p (Fig 4A). Next, we performed dual-luciferase reporter assay, RIP assay and RNA pull down assay to confirm the direct binding between lncRNA-ATB and miR-141-3p in A549 cells. As shown in Fig 4B, the luciferase activity of ATB-WT was notably inhibited by transfection with miR-141-3p mimics but no obvious effect was observed in the luciferase activity of ATB-Mut following overexpression of miR-141-3p. For RIP assay, lncRNA-ATB and miR-141-3p were visibly enriched by Ago2 antibody relative to control IgG antibody (*P* < 0.05, Fig 4C). RNA pull-down data revealed that lncRNA-ATB enrichment in miR-141-3p-Bio immunoprecipitates was markedly higher than that in NC-Bio immunoprecipitates (*P* < 0.05, Fig 4D). All in all, these data indicated that lncRNA-ATB is a target of miR-141-3p.

### Negative correlation between miR-141-3p and lncRNA-ATB in human NSCLC tissues

**Fig 5:**
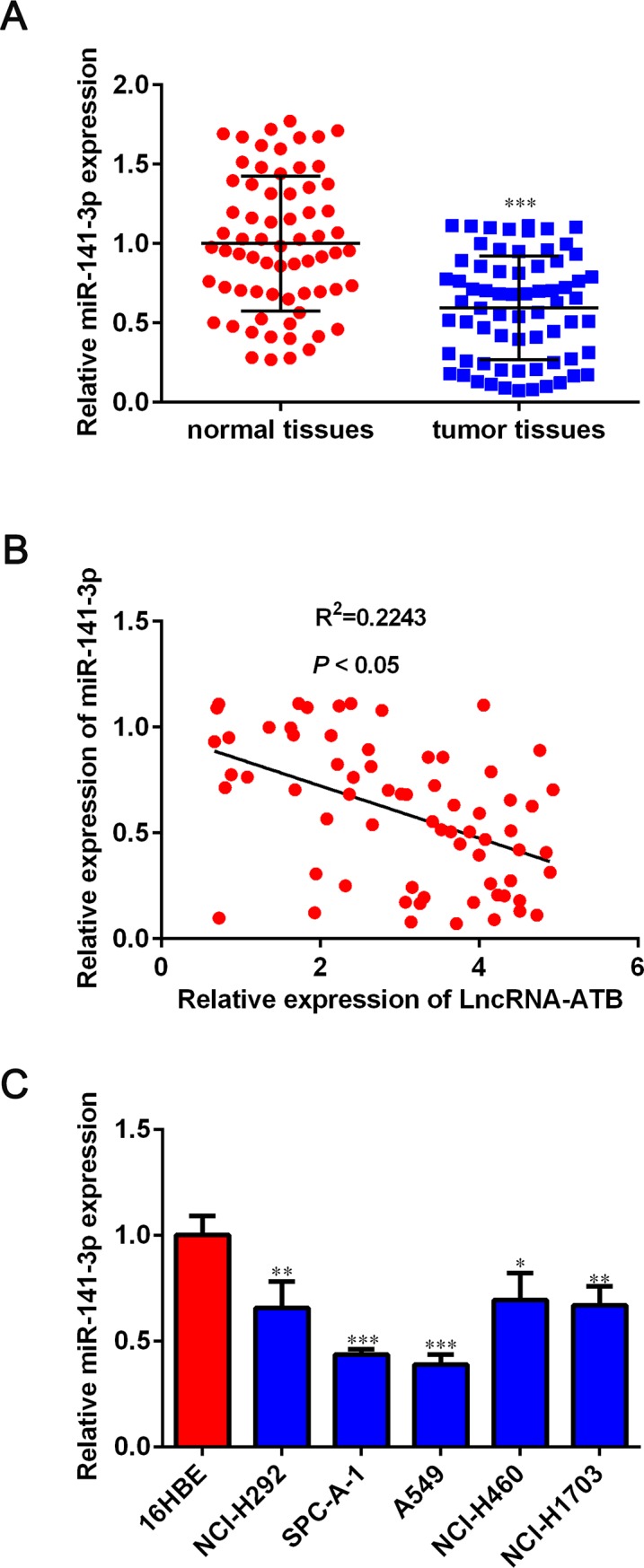
Expression of miR-141-3p in NSCLC tissues and its association with lncRNA-ATB. A. MiR-141-3p expression in para-carcinoma tissues and NSCLC tissues was examined by qRT-PCR. B. The expression relationship between lncRNA-ATB and miR-141-3p in NSCLC tissues was analyzed by Spearman’s correlation analysis. C. MiR-141-3p expression in NSCLC cell lines and normal 16HBE cell line was detected using qRT-PCR. Data are presented as mean ± SD. *P < 0.05, **P < 0.01 and ***P < 0.001 versus normal tissue group or 16HBE group.

We further detected the expression level of miR-141-3p in NSCLC tissues and cell lines. As shown in Fig 5A, miR-141-3p expression was lower in tumor tissues than that in normal tissues. And we have analyzed the relationship between miR-141-3p and lncRNA-ATB expression levels in NSCLC tissues. Data in Fig 5B suggested that lncRNA-ATB expression was inversely correlated with miR-141-3p expression level in NSCLC tissues. In addition, all five NSCLC cell lines had lower miR-141-3p expression than the normal 16HBE cell line (*P* < 0.05, Fig 5C). These data suggested that lncRNA-ATB suppresses miR-141-3p expression in NSCLC.

### LncRNA-ATB promotes tumor growth in vivo

**Fig 6:**
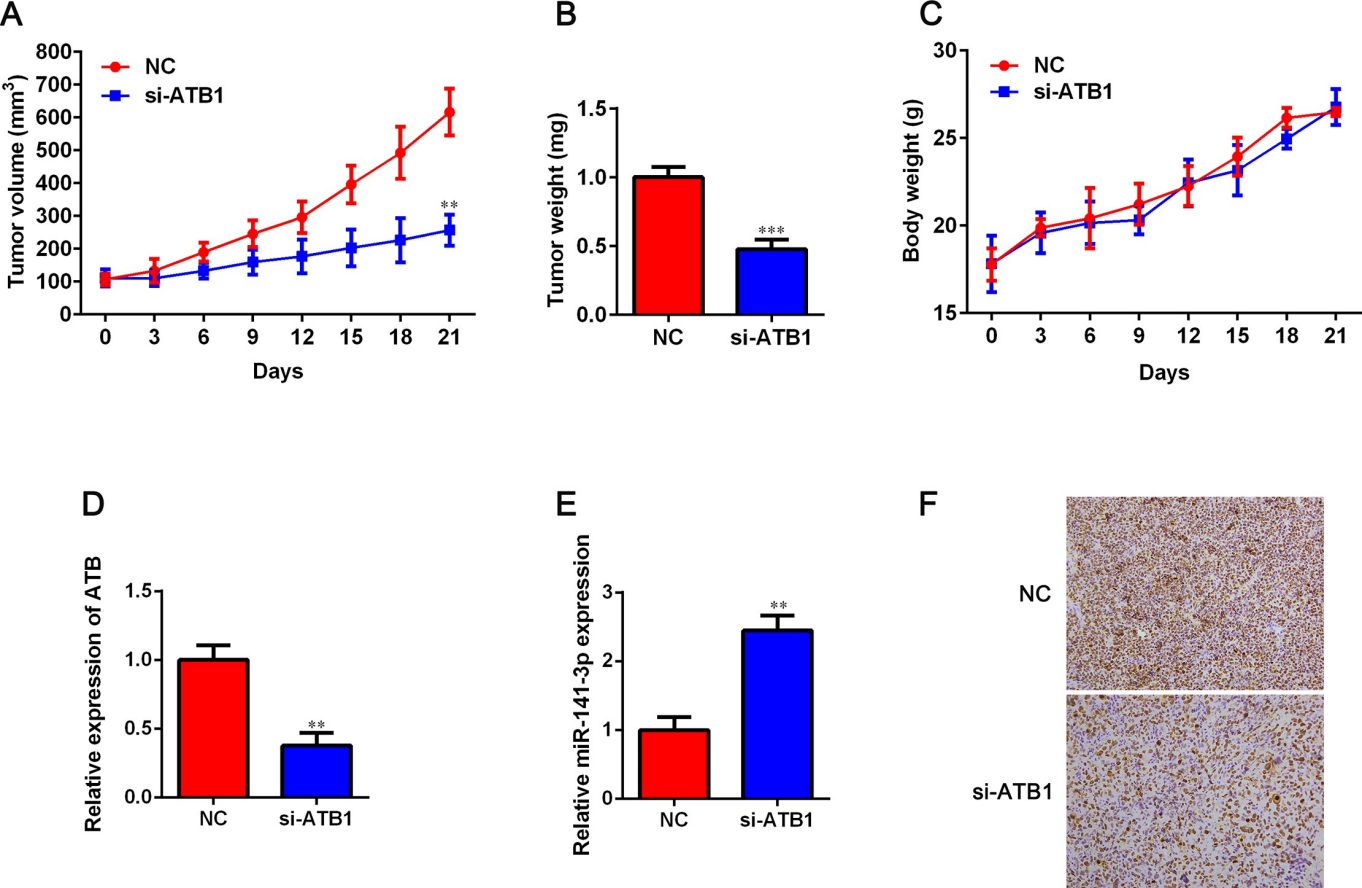
Effect of lncRNA-ATB on tumor growth *in vivo*. A. In mice injected with A549 cells, the tumor size was lower in the si-lncRNA-ATB nude mice than in the si-NC group (*P* < 0.05). B. Tumors were harvested on the day 27 and weighed. C. Body weight was detected. D. The relative expression of lncRNA-ATB was detected in tumors. E. miR-141-3p expression in tumors was determined by using qRT-PCR. F. Immunostaining analysis was conducted to examine the positive rate of Ki67 in tumors derived from si-lncRNA-ATB transfected A549 cells. Data are represented as mean ± SD. **P < 0.01 and ***P < 0.001 versus NC group.

To further confirm the oncogenic activity of lncRNA-ATB, we inoculated A549 cells transfected with si-lncRNA-ATB or si-NC into nude mice. As shown in Fig 6A and 6B, tumor volume and weight in si-lncRNA-ATB group were significantly lower than those in si-NC group. However, there was no difference between the si-NC and si-lncRNA-ATB groups (Fig 6C). Compared with si-NC group, si-lncRNA-ATB group had a significant decrease in lncRNA-ATB expression and a notable increase in miR-141-3p expression (*P* < 0.05, Fig 6D and 6E). In addition, we observed that the positive rate of Ki67 in the si-lncRNA-ATB group was lower than that in the si-NC group (Fig 6F). Taken together, these results indicated that that lncRNA-ATB exerted its oncogenic activity by targeting miR-141-3p in NSCLC.

## Discussion

In recent years, more and more lncRNAs are found to be up-regulated in NSCLC compared with normal tissues, suggesting that lncRNAs can be considered, at least for NSCLC, as therapeutic targets. Hence, exploration of more lncRNAs is necessary for efficiently enhancing the early detection of NSCLC patients.

LncRNA-ATB, a novel lncRNA activated by TGF-β, has been identified as an oncogene. Previous studies have revealed that lncRNA-ATB facilitates osteosarcoma cell growth, mobility and invasive ability through inhibiting miR-200s [13]. Xiong et al. found that higher expression of lncRNA-ATB was observed in renal cell carcinoma tissues and renal cancer cell lines than that in adjacent normal kidney tissues and normal human proximal tubule epithelial cell line HK-2. Silencing lncRNA-ATB could suppress renal cell growth, induce apoptosis, reverse epithelial-to-mesenchymal transition and inhibit cell mobility and invasion [14]. In colon cancer, striking differences were observed in overall survival and disease-free survival in cases with both high lncRNA-ATB expression and low E-cadherin expression, suggesting that lncRNA-ATB mediated E-cadherin inhibition contributes to colon cancer development and progression and indicates poor prognosis [15]. Study by Yuan et al found that up-regulation of lncRNA-ATB was detected in hepatocellular carcinoma (HCC) and predicted poor prognosis. LncRNA-ATB induced EMT and promoted metastasis of HCC cell lines by sponging miR-200 family [16]. The role of lncRNA-ATB was also investigated in other cancer cell lines, such as prostate cancer [17], glioma [18], cervical cancer [19] and breast cancer [20]. However, the value of lncRNA-ATB in clinical practice of NSCLC is unknown.

In the current study, we attempted to explore the functional role of lncRNA-ATB in NSCLC. We first demonstrated that the expression level of lncRNA-ATB was significantly up-regulated in NSCLC tissues. We further explored the relationships between lncRNA-ATB expression levels and clinicopathological characteristics. The levels of lncRNA-ATB were positively correlated with tumor size, histological grade and tumor metastasis in patients with NSCLC. In order to explore the role of lncRNA-ATB on NSCLC cell biological function, loss-of function experiments were performed. Our results demonstrated that down-expression of lncRNA-ATB inhibited proliferation, colony formation migration and invasion of NSCLC cells. EMT can be observed by detecting the epithelial markers E-cadherin and mesenchymal markers, such as Vimentin and N-cadherin [21]. Remarkably, we found that after the knockdown of lncRNA-ATB, some hallmarks of the mesenchymal cells were reduced, whereas the epithelial protein E-cadherin was increased, indicating that lncRNA-ATB could facilitate metastasis through EMT.

Increasing evidences have shown that miR-141-3p functions as a tumor suppressor. MiR-141-3p could suppress colorectal cancer cell growth and metastasis via targeting TRAF5 [22]. Wang et al revealed that up-regulation of miR-141-3p led to a significant repression of proliferation and induction of apoptosis in osteosarcoma cells [23]. Lower expression of miR-141-3p was observed in bone metastatic prostate cancer tissues than that in non-bone metastatic prostate cancer tissues, suggesting that down-regulation of miR-141-3p could facilitate bone metastasis in prostate cancer [24]. In NSCLC, Li et al found that miR-141-3p was markedly decreased in NSCLC tissues compared with adjacent normal tissues and low expression of miR-141-3p predicted poor overall survival [25]. Consistently, our findings demonstrated that miR-141-3p was inversely associated with lncRNA-ATB expression. According to the competing endogenous RNA (ceRNA) hypothesis, lncRNAs functioned in multiple biological processes through acting as ceRNAs to regulate microRNAs (miRNAs) [26–28]. we proposed that lncRNA-ATB might act as a ceRNA to regulate relevant cellular control. Therefore, we adopted bioinformatics analysis and luciferase assays to verify the potential target of lncRNA-ATB. Interestingly, subsequent luciferase reporter experiments, RNA pull-down analysis, RIP and qRT-PCR assay confirmed that lncRNA-ATB suppressed the expression of miR-141-3p via direct interaction.

In summary, for the first time, we have described the clinicopathologic features as well as the critical function of lncRNA-ATB in NSCLC cell growth, migration, invasion and EMT. We also indicated miR-141-3p was the target of lncRNA-ATB. These results may open new prospects for targeting lncRNA-ATB in the treatment of NSCLC patients.

## Supporting information

S1 Fig Knockdown of lncRNA-ATB expression by using a second si-lncRNA-ATB inhibited growth in NSCLC cells. A, B. Cell growth was measured by the CCK-8 assay after A549 and SPC-A-1 cells after transfected with si- ATB2. C, D. Colony formation assay was performed to detect the proliferation ability of A549 and SPC-A-1 cells after transfected with si-ATB2. E, F. Western blot assay showed the protein level of EMT markers in A549 and SPC-A-1 cells after transfected with si-ATB2. Data are expressed as mean ± SD. **P < 0.01 versus NC group.
(TIF)

S2 Fig Effects of lncRNA-ATB on migration and invasion in NSCLC cells. A, B. Detection for cell migration ability of A549 and SPC-A-1 cells after transfected with si-ATB2. C, D. Transwell chamber assay was employed to examine the invasion ability of A549 and SPC-A-1 cells after transfected with si-ATB2. Data are presented as mean ± SD. **P < 0.01 versus NC group.
(TIF)

S1 Raw images (PDF)
