# Supplementary figures and images for: Long non-coding RNA ATB promotes human non-small cell lung cancer proliferation and metastasis by suppressing miR-141-3p
Source: PLoS One. 2020 Feb 24;15(2):e0229118. doi: 10.1371/journal.pone.0229118 (PMC7039450; doi:10.1371/journal.pone.0229118)

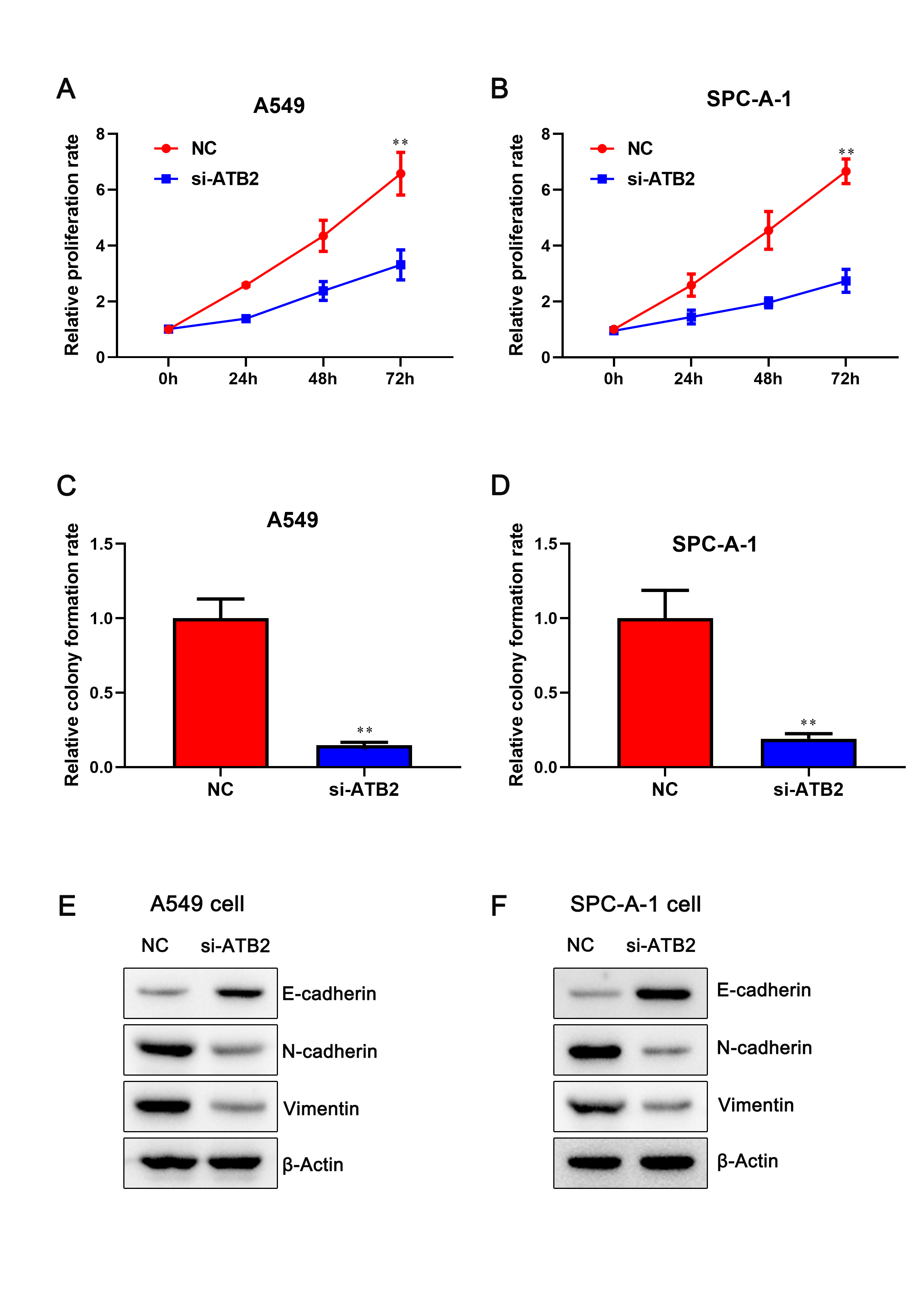

Supplement: S1 Fig — A, B. Cell growth was measured by the CCK-8 assay after A549 and SPC-A-1 cells after transfected with si- ATB2. C, D. Colony formation assay was performed to detect the proliferation ability of A549 and SPC-A-1 cells after transfected with si-ATB2. E, F. Western blot assay showed the protein level of EMT markers in A549 and SPC-A-1 cells after transfected with si-ATB2. Data are expressed as mean ± SD. **P < 0.01 versus NC group. (TIF) [file pone.0229118.s001.tif]

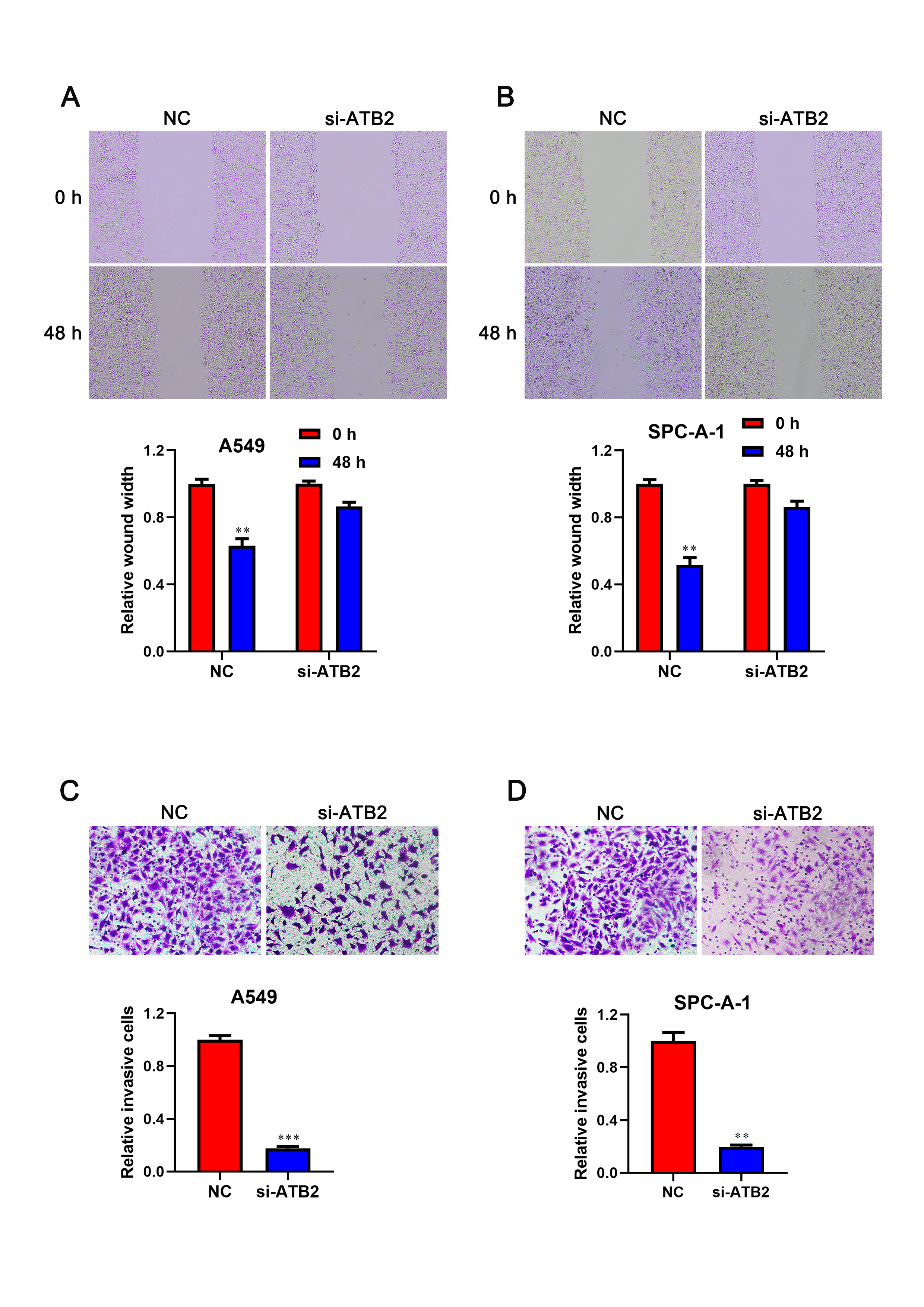

Supplement: S2 Fig — A, B. Detection for cell migration ability of A549 and SPC-A-1 cells after transfected with si-ATB2. C, D. Transwell chamber assay was employed to examine the invasion ability of A549 and SPC-A-1 cells after transfected with si-ATB2. Data are presented as mean ± SD. **P < 0.01 versus NC group. (TIF) [file pone.0229118.s002.tif]
