# Supplementary material for: Long non-coding RNA ATB promotes human non-small cell lung cancer proliferation and metastasis by suppressing miR-141-3p
Source: PLoS One. 2020 Feb 24;15(2):e0229118. doi: 10.1371/journal.pone.0229118 (PMC7039450; doi:10.1371/journal.pone.0229118)

Fig. 3E E-cadherin

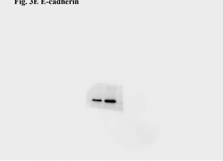

Fig. 3E N-cadherin

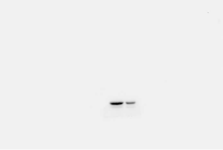

Fig. 3E N-cadherin overexposure

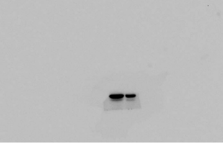

Fig. 3E Vimentin

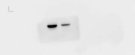

Fig. 3E β-actin

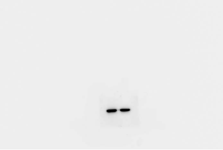

Fig. 3F E-cadherin

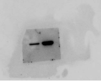

Fig. 3F N-cadherin

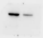

Fig. 3F Vimentin

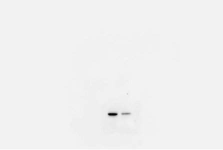

Fig. 3F β-actin

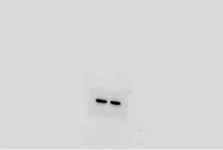

Supplementary Fig.1E E-cadherin

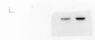

Supplementary Fig.1E N-cadherin

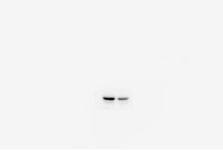

Supplementary Fig.1E N-cadherin overexposure

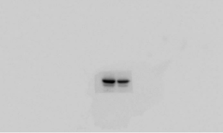

Supplementary Fig.1E Vimentin

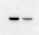

Supplementary Fig.1E β-actin

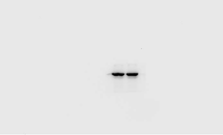

Supplementary Fig.1F E-cadherin

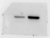

Supplementary Fig.1F N-cadherin

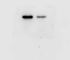

Supplementary Fig.1F N-cadherin overexposure

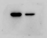

Supplementary Fig.1F Vimentin

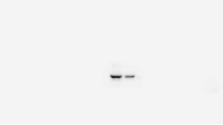

Supplementary Fig.1F Vimentin overexposure

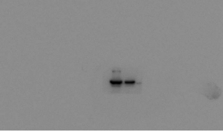

Supplementary Fig.1F β-actin

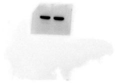

Supplement: S1 Raw images — (PDF) [file pone.0229118.s003.pdf]
